# Deletion of angiotensin II type 1 receptor gene or scavenge of superoxide prevents chronic alcohol-induced aortic damage and remodelling

**DOI:** 10.1111/j.1582-4934.2012.01569.x

**Published:** 2012-09-26

**Authors:** Yang Bai, Yi Tan, Bo Wang, Xiao Miao, Qiang Chen, Yang Zheng, Lu Cai

**Affiliations:** a The Cardiovascular Center, the First Hospital of Jilin UniversityChangchun, China; b Department of Pediatrics, University of LouisvilleKY, USA; c Chinese-American Research Institute for Diabetic Complications, Wenzhou Medical CollegeWenzhou, Zhejiang, China; d The Second Hospital of Jilin UniversityChangchun, China; e School of Public Health, Jilin UniversityChangchun, China

**Keywords:** Alcoholic vascular damage, Nitrosative damage, Angiotensin II, AT1, Superoxide

## Abstract

To investigate whether chronic alcohol consumption induces vascular injury via angiotensin II (Ang II) type 1 (AT1) receptor-dependent superoxide generation, male transgenic mice with knockout of AT1 gene (AT1-KO) and age-matched wild-type (WT) C57BL/6 mice were pair-fed a modified Lieber-DeCarli alcohol or isocaloric maltose dextrin control liquid diet for 2 months. Ethanol content (%, W/V) in the diet was 4.8 (34% of total calories) at initiation, and gradually increased up to 5.4 (38% of total calories). For some WT mice with and without alcohol treatment, superoxide dismutase mimetic (MnTMPyP) was given simultaneously by intraperitoneal injection at 5 mg/kg body weight daily for 2 months. At the end of studies, aortas were harvested for histopathological and immunohistochemical examination. Significant increases in the wall thickness and structural disarrangement of aorta were found in alcohol group, along with significant increases in aortic oxidative and/or nitrosative damage, expressions of NADPH oxidases (NOXs), inflammatory response, cell death and proliferation, and remodelling (fibrosis). However, these pathological changes were completely attenuated in alcohol-treated AT1-KO mice or in alcohol-treated WT mice that were also simultaneously treated with MnTMPyP for 2 months. These results suggest that chronic alcohol consumption may activate NOX via Ang II/AT1 receptor, to generate superoxide and associated peroxynitrite that in turn causes aortic nitrosative damage, inflammation, cell death and proliferation, and remodelling. Therefore, blocking Ang II/AT1 system or scavenging superoxide may become a potential preventive and/therapeutic approach to alcoholic vascular damage.

## Introduction

Alcoholic abuse remains a major public health problem in the United States, affecting more than 20 million individuals, leading to loss of 100,000 lives annually [1, 2]. Chronic high-dose alcoholic consumption most commonly causes hepatic, gastrointestinal, nervous and cardiovascular injuries [3]. Reportedly chronic alcoholic consumption is also associated with an increased incidence of various cardiovascular diseases (CVD) [4–7]. Although molecular mechanisms by which alcohol causes vascular injury remain elusive, oxidative stress and damage has been proposed to play a critical role in the pathogenesis of alcoholic vascular injuries [8, 9]. In addition, systemic and organ local renin-angiotensin systems were found to be increased in parallel with alcohol-induced oxidative damage and vascular injury [1–7].

It is known that angiotensin II (Ang II) stimulates superoxide generation via its type 1 (AT1) receptor to activate NADPH oxidase (NOX) in the vascular wall [10]. Seven NOX family members (i.e., NOX 1–5, Duox 1 and Duox 2) have been identified [11], of which NOX1, NOX2 and NOX4 are the main isoforms expressed in cardiovascular cells [12]. Each of these isoforms exists as a heterodimer with a lower molecular weight p22^phox^ subunit and is predicted to be membrane-bound but there are several major differences between the isoforms. NOX2 is normally quiescent and is acutely activated by stimuli such as G-protein-coupled receptor agonists (e.g., Ang II), growth factors, and cytokines in a tightly regulated process [11]. For instance, NOX2 activation requires stimulus-induced membrane translocation of p47^phox^ (i.e. formation of the active oxidase complex at the membrane) [13]. We and others have demonstrated that activation of p47^phox^ by diabetes and Ang II led to significant cardiac oxidative damage and cell death, and consequently resulted in cardiomyopathy [14, 15]. Unlike NOX2, NOX4 does not have a requirement for additional regulatory subunits. NOX4 has constitutive low-level activity and seems to be regulated largely by changes in abundance [11].

Superoxide production through NOX activation by Ang II has been associated with the development of endothelial dysfunction and atherosclerosis. For instance, hypercholesterolaemia was associated with AT1-receptor up-regulation, endothelial dysfunction, and increased NOX-dependent vascular superoxide production [16]. The improvement of endothelial dysfunction, inhibition of the NOX, and reduction of early plaque formation by an AT1-receptor antagonist, Bay 10-6734, suggests a crucial role of Ang II-mediated superoxide production in the pathogenesis of atherosclerosis [16]. Another study demonstrated that male rats that were treated with 20% alcohol daily for 12 weeks exhibited increased aortic inflammation [tumour necrosis factor (TNF)-α and MCP-1 protein expressions] and elevated Ang II levels as compared with control rats. Aortic NOX activity, membrane and cytosolic subunits p22^phox^ and p47^phox^ expressions were significantly increased whereas Cu/Zn-SOD activity and protein expression significantly decreased in alcohol-treated group compared to control [17]. These results suggest the possibility for Ang II via its AT1 receptor to activate NOX-mediated oxidative damage that in turn causes aortic inflammation and remodelling. However, there remains little or no direct evidence to indicate the direct role of AT1 in such pathological changes by gene manipulation.

The present study, therefore, was to investigate the relationship of chronic alcohol-induced oxidative stress/damage with vascular inflammation and remodelling in the mouse model. To directly define the critical role of AT1 in alcohol-induced vascular cell death and remodelling, transgenic mice with knockout of AT1 gene (AT1-KO) were used for chronic alcohol feeding, and to define the causative role of NOX-derived superoxide in these pathogenic changes, chronic alcohol-fed mice were simultaneously treated with superoxide dismutase mimetic to scavenge superoxide. Both animal models exhibited completely resistant to alcoholic induction of aortic oxidative stress/damage, cell death, inflammation, and remodelling. Therefore, this study provides the direct evidence that Ang II/AT1 receptor system plays a pivotal role in chronic alcohol consumption-induced aortic oxidative and/or nitrative damage, cell death, inflammation, and remodelling, which is most likely mediated by NOX-mediated superoxide generation.

## Materials and methods

### Animals

AT1-KO mice and their wild-type (WT) C57BL/6 mice were purchased from Harlan Laboratories (Indianapolis, IN). Mice were treated according to experimental procedures approved by the Institutional Animal Care and Use Committee of the University of Louisville, which is compliant with the Guide for the Care and Use of Laboratory Animals published by the US National Institutes of Health (NIH Publication No. 85–23, revised 1996).

Four month-old male of two strain mice were pair-fed modified Lieber-DeCarli alcohol or isocaloric maltose dextrin control liquid diet for 2 months with a stepwise feeding procedure as used before [18, 19]. We used this procedure because a long-term feeding of mice with the Lieber-DeCarli ethanol liquid diet causes decreases in food intake and body weight gain, while the stepwise feeding procedure can eliminate the possible confounding of malnutrition caused by decreased food intake [18, 19]. The ethanol content (%, w/v) in the diet was 4.8 (34% of total calories) at initiation, and gradually increased up to 5.4 (38% of total calories). The amount of food given to the pair-fed mice was matched to that of the alcohol-fed mice measured on the previous day. In a separate study, WT mice with and without alcohol feeding were given superoxide dismutase mimetic MnTMPyP [Mn (111) tetrakis 1-methyl 4-pyridylporphyrin pentachloride, purchased from Sigma Chemical Co, St. Louis, MO] by intraperitoneal injection at 5 mg/kg body weight daily for 2 months. Because MnTMPyP was dissolved in PBS, mice serving as vehicle controls were given the same volume of PBS. At the end of experiments, all mice were anaesthetized with 2, 2, 2-tribromoethanol (Sigma, St. Louis, MO) at the dose of 240 mg/kg body weight for killing and collecting aortas.

### Aorta preparation and histopathological examination

After anaesthesia, thorax was opened and descending thoracic aortas were isolated carefully and cleaned of surrounding fat and connective tissue. Aortas tissues were fixed in 10% buffered formalin and then cut into ring segments (2–3 mm in length) for being dehydrated in graded alcohol series, cleared with xylene, embedded in paraffin and sectioned at 5 μm thickness for pathological and immunohistochemical staining.

Paraffin sections (5 μm thickness) from aortic tissues were dewaxed, followed by incubation with 1X Target Retrieval Solution (Dako, Carpinteria, CA) in a microwave oven for 15 min at 98°C for antigen retrieval, then with 3% hydrogen peroxide for 15 min at room temperature and with 5% animal serum for 30 min, respectively. These sections were incubated with primary antibodies [connective tissue growth factor (CTGF) and transforming growth factor (TGF)-β1 at 1:100 dilution, Santa Cruz Biotechnology, CA; 3-nitrotyrosine (3-NT) at 1:400 dilution, Millipore, CA; 4-Hydroxy-2-nonenal (4-HNE) at 1:400 dilution, Alpha diagnostic international, TX; plasminogen activator inhibitor-1 (PAI-1) at 1:100 dilution, BD Bioscience, CA; TNF-α at 1:100 dilution, Cell Signaling, MA; Ki67 at 1:300 dilution, Abcam, CA; p47^phox^ at 1:100 dilution, Life Span BioSciences, CA; NOX4 at 1:200 dilution, Novus Biologicals, CO] overnight at 4°C. After sections were washed with PBS, they were incubated with horseradish peroxidase conjugated secondary antibodies (1:300 - 400 dilutions with PBS) for 2 hrs in room temperature. For the development of colour, sections were treated with peroxidase substrate DAB kit (Vector Laboratories, Inc. Burlingame, CA) and counterstained with haematoxylin.

### Sirius-red staining for collagen

Aortic fibrosis was reflected by Sirius-red staining for collagen as described in our previous study [20]. Briefly, 5 μm tissue sections were used for Sirius-red staining with 0.1% Sirius-red F3BA and 0.25% Fast Green FCF. Sections stained for Sirius-red then were assessed for the proportion of collagen as fibrosis using a Nikon Eclipse E600 microscopy system.

### Terminal transferase dUTP nick end labelling staining

For detecting apoptotic cell death in the aorta, we performed the terminal transferase dUTP nick end labelling (TUNEL) staining by using ApopTag Plus Peroxidase *In Situ* Apoptosis Detection Kit (Chemicon, Temecula, CA) according to the manufacturer's instructions. Mouse testicular tissue was used as a positive control. Cells with TUNEL-positive nuclei were counted under high magnification (40X) in five random fields for each of two slides from each mouse, and presented as TUNEL-positive nuclei per 100 vascular cell nuclei.

### Real-time qPCR

Collected aortas were snap frozen in liquid nitrogen and kept at - 80°C. Total RNA was extracted using the TRIzol Reagent (Invitrogen, USA). RNA concentrations and purities were quantified using a Nanodrop ND-1000 spectrophotometer. First-strand complimentary DNA (cDNA) was synthesized from total RNA according to manufacturer's protocol from the RNA PCR kit (Promega, Madison, WI). Reverse transcription was performed with 0.5 μg of total RNA in 12.5 μl of the solution containing 4 μl 25 mM MgCl_2_, 4 μl AMV reverse transcriptase 5 X buffer, 2 μl dNTP, 0.5 μl RNase inhibitor, 1 μl of AMV reverse transcriptase and 1 μl of oligo dT primer, which were added with nuclease-free water to make a final volume of 20 μl.

Reaction system was run at 42°C for 50 min and 95°C for 5 min. Primers [AT1: Mm00616371_m1, CTGF: Mm01192933_g1, TGF-β1 Mm00441724_m1, β-actin: Mm00607939_s1] for PCR were purchased from Applied Biosystems (Carlsbad, CA, USA).

Real-time qPCR (quantitative PCR) was carried out in a 20 μl reaction buffer that included 10 μl of TaqMan Universal PCR Master Mix, 1 μl of primer, 9 μl of cDNA with the ABI 7300 Real-Time PCR system. The fluorescence intensity of each sample was measured at each temperature change to monitor amplification of the target gene. The comparative cycle time (CT) was used to determine fold differences between samples.

### Statistical Analysis

Data were collected from several animals (n ≥ 4) and presented as means±SD. We used Image Pro Plus 6.0 software and a IOD (integrated optical density) divided area method to identify the positive staining area of interest. Comparisons were performed by two-way ANOVA for the different groups, followed by post hoc pairwise repetitive comparisons using Tukey's test with Origin 7.5 Lab data analysis and graphing software. Statistical significance was considered as *P* < 0.05.

## Results

### Alcohol up-regulated AT1 mRNA expression in the aorta of WT mice

**Fig 1:**
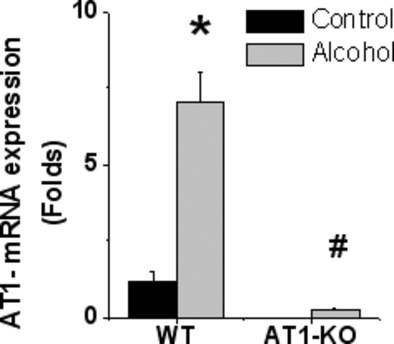
AT1 mRNA expression in WT mice, but not in AT1-KO mice. AT1-KO and WT mice were fed alcohol for 2 months and then aortic tissues were collected for measuring the AT1 mRNA expression with real-time qPCR. Data are presented as means ± SD (WT control: n = 5; WT alcohol: n = 8; AT1-KO control: n = 4; AT1-KO alcohol: n = 4). *, *P* < 0.05 vs control; #, *P* < 0.05 vs WT alcohol group.

For the first study AT1-KO mice and age-matched WT mice were fed with alcohol or isocaloric maltose dextrin control liquid diet for 2 months. Real-time qPCR analysis revealed that AT1 mRNA expression was detectable in WT control mice and significantly increased in alcohol-treated WT mice, but not in AT1-KO mice (Fig. 1).

### AT1-KO mice were resistant to alcohol-induced aortic pathological changes

**Fig 2:**
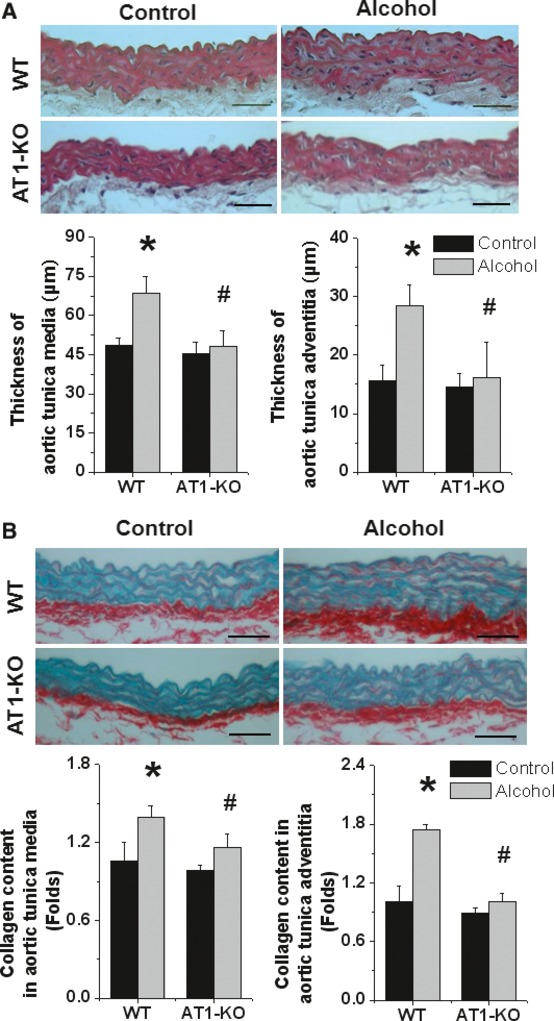
AT1-KO mice are resistant to alcohol-induced aortic pathological changes. (A) H&E staining indicates the thickness increase and structural disarrangement in both aortic tunica media and adventitia of alcohol-fed WT mice but not AT1-KO mice. (B) Sirius-red staining revealed increased collagen accumulation in both aortic tunica media and adventitia of alcohol-fed WT mice but not AT1-KO mice. Data are presented as means ± SD (the animal number for each group is indicated in Figure 1). *, *P* < 0.05 vs corresponding control; #, *P* < 0.05 vs WT alcohol group. Bar = 50 μM.

Pathological examination with haematoxylin-eosin (H&E) staining indicated that alcohol induced aortic wall thickness increase and structural disarrangement in both tunica media and adventitia of WT mice (Fig. 2A). However, these pathological changes were not evident in alcohol-treated AT1-KO mice, suggesting that AT1 receptor is required for alcohol-induced pathological changes in aortas.

**Fig 3:**
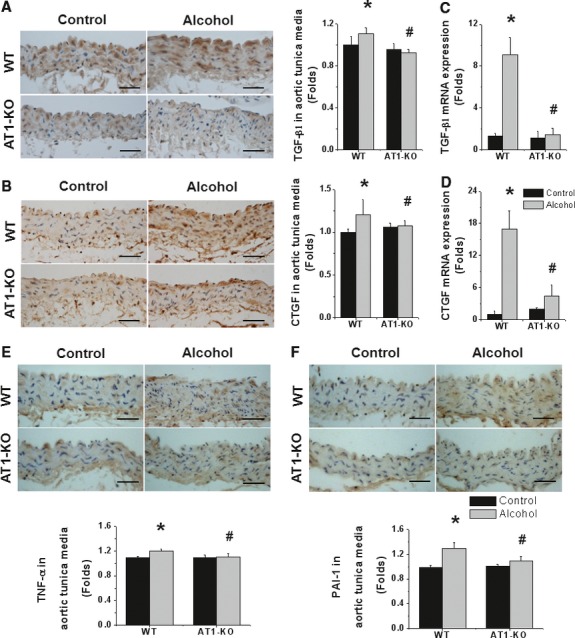
AT1-KO mice are resistant to alcohol-induced aortic fibrosis and inflammation. Immunohistochemical staining showed increased expressions of TGF-β1 at protein (A) and mRNA (C) levels and CTGF at protein (B) and mRNA (D) levels as index of fibrosis, and TNF-α (E) and PAI-1 (F) at protein level as index of inflammation. Data are presented as means ± SD (the animal number for each group is indicated in Figure 1). *, *P* < 0.05 vs corresponding control; #, *P* < 0.05 vs WT alcohol group. Bar = 50 μM.

To further detect aortic remodelling (fibrosis), Sirius-red staining was performed and it showed that alcohol induced an obvious collagen accumulation in both aortic tunica media and adventitia in WT mice but not in AT1-KO mice (Fig. 2B). Induction of aortic fibrosis was further confirmed by immunohistochemical staining of two molecular mediators of fibrosis TGF-β1 (Fig. 3A) and CTGF (Fig. 3B). Real-time qPCR analysis also showed significant increases in mRNA expressions of TGF-β1 (Fig. 3C) and CTGF (Fig. 3D). There was no significant increase in TGF-β1 or CTGF either at protein or mRNA level in the aorta of alcohol-treated AT1-KO mice (Fig. 3A-D).

Because inflammatory response has been suggested to play an important role in alcohol-induced aortic pathogenesis, inflammatory mediators, TNF-α (Fig. 3E) and PAI-1 (Fig. 3F), were examined by immunohistochemical method. Both inflammatory mediators were significantly increased in the aorta of alcohol-treated WT mice, but not in AT1-KO mice.

**Fig 4:**
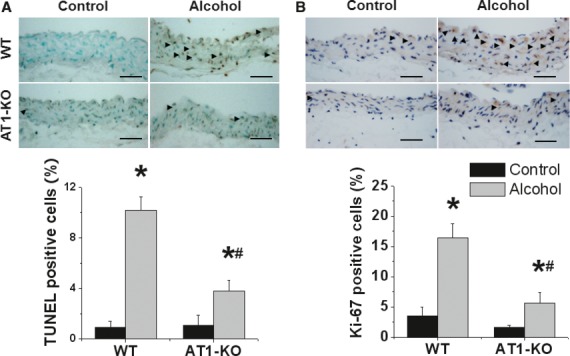
AT1-KO mice are resistant to alcohol-induced aortic cell death and proliferation. Apoptotic cell death was detected by TUNEL staining (A), arrows indicating TUNEL-positive cells in both aortic tunica intima and media. Cell proliferation was detected by Ki67 immunohistochemical staining (B), arrows indicating positive Ki67 cells. Data are presented as means ± SD (the animal number for each group is indicated in Figure 1). *, *P* < 0.05 *vs* corresponding control; #, *P* < 0.05 vs WT alcohol group. Bar = 50 μM.

Next whether or not alcohol induces apoptosis and proliferation in the aorta, and if so, whether or not AT1-KO mice are resistant to these effects were examined. Apoptotic cells, detected by TUNEL staining (Fig. 4A), were showed that alcohol induced a significant increase in the incidence of apoptotic cells. In addition, immunohistochemical staining of Ki67 showed that alcohol also induced a significant increase in aortic cell proliferation (Fig. 4B). However, both aortic cell death and proliferation were not seen in the aorta of alcohol-treated AT1-KO mice (Fig. 4).

**Fig 5:**
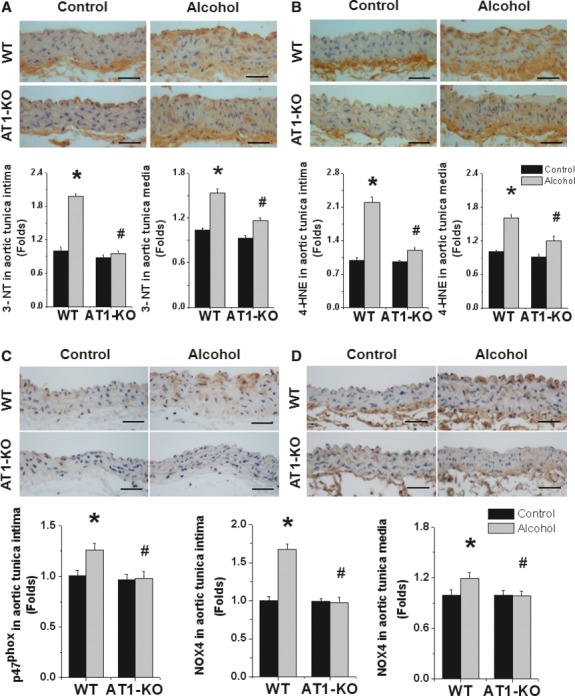
AT1-KO mice are resistant to alcohol-induced oxidative stress. Oxidative stress was examined by immunohistochemical staining of 3-NT (A) and 4-HNE (B). NOX2 (C) and NOX4 (D) expression was examined by immunohistochemical staining. Data are presented as means ± SD (the animal number for each group is indicated in Figure 1). *, *P* < 0.05 *vs* corresponding control; #, *P* < 0.05 *vs* WT alcohol group. Bar = 50 μM.

Reportedly oxidative stress plays a critical role in alcoholic aortic injury, we next examined whether or not alcohol induced oxidative damage in the aorta by examining nitrosative damage, indexed by 3-NT (Fig. 5A), and lipid peroxidation, indexed by 4-HNE (Fig. 5B) with immunohistochemical staining. Both 3-NT and 4-HNE accumulations were significantly increased in both aortic tunica intima and media of alcohol-treated WT mice, but not in AT1-KO mice (Fig. 5).

To investigate whether or not aortic NOX expression increases in response to alcohol treatment, we examined p47^phox^ (NOX2, Fig. 5C) and NOX4 (Fig. 5D) expressions with immunohistochemical staining, which indicates that alcohol increased both p47^phox^ and NOX4 expressions predominantly in aortic tunica intima, which is consistent with 3-NT and 4-HNE accumulation predominantly in tunica intima (Fig. 5A,B). There was slight increased expression of NOX4 in tunica media (Fig. 5D). Expression of p47^phox^ was mainly located in aortic tunica intima whereas there was no significant expression of p47^phox^ in tunica media. No significant increase in either p47^phox^ or NOX4 expression in alcohol-treated AT1-KO mice (Fig. 5).

### Treatment of alcohol-fed mice with superoxide dismutase mimetic completely prevented alcohol-induced aortic pathogenic changes

**Fig 6:**
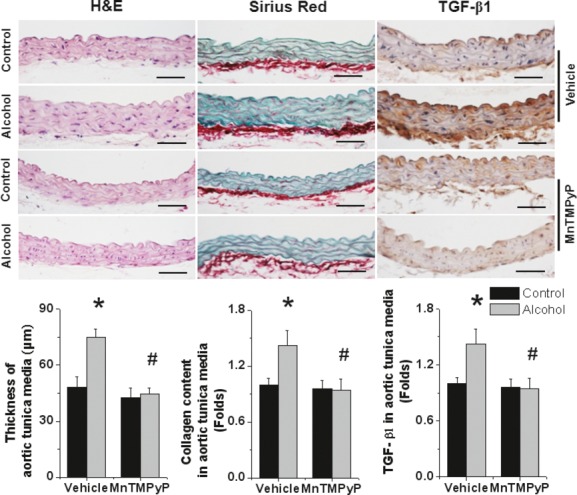
Treatment with MnTMPyP prevents alcohol-induced aortic morphology changes. WT mice with and without 2-month alcohol feeding were simultaneously treated with superoxide dismutase mimetic MnTMPyP at 5 mg/kg body weight daily for 2 months. Mice serving as vehicle controls were given the same volume of PBS. Aortic tissues were stained with H&E, Sirius-red, and TGF-β1, as indicated with semi-quantitative analysis. Data were presented as means ± SD (Vehicle control: *n* = 8; Vehicle alcohol: n = 6; MnTMPyP: *n* = 5; MnTMPyP alcohol: n = 7). *, *P* < 0.05 *vs* corresponding control; #, *P* < 0.05 *vs* vehicle alcohol group. Bar = 50 μM.

The above study clearly showed the important role of AT1 receptor in alcohol-induced aortic pathogenesis. To further define the role of NOX-generated superoxide and peroxynitrite activated by Ang II/AT1 in alcohol-induced aortic pathogenesis, we have treated alcohol-fed mice simultaneously with superoxide dismutase mimetic MnTMPyP at 5 mg/kg body weight daily for 2 months. Pathological changes of aortas were examined with H&E staining, which indicates that alcohol-induced aortic wall thickness increase and structural disarrangement in alcohol-fed mice were completely prevented by scavenging superoxide generation with superoxide dismutase mimetic MnTMPyP in alcohol-fed mice with MnTMPyP (Fig. 6). Sirius-red staining for collagen and immunohistochemical staining for TGF-β1 (Fig. 6) depicted that alcohol induced fibrotic responses in aortic tunica media were also completely attenuated by MnTMPyP treatment.

**Fig 7:**
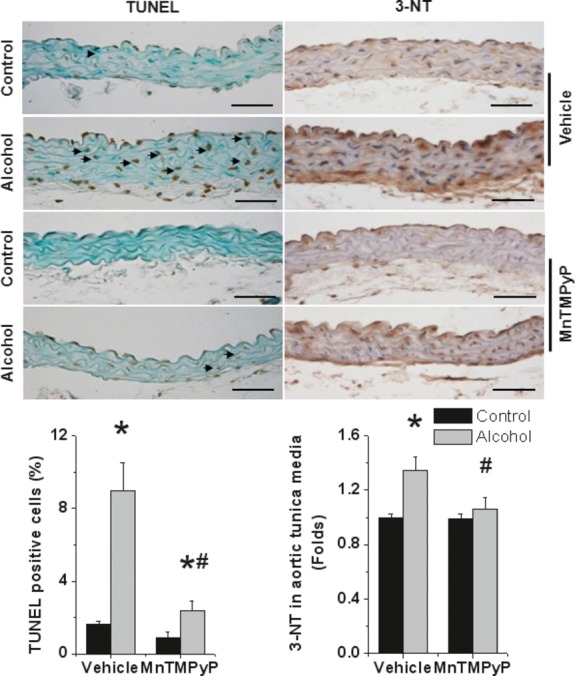
Treatment with MnTMPyP prevents alcohol-induced aortic cell death and oxidative damage. Aortic tissues were subjected to TUNEL staining for detecting apoptotic cell death in both aortic tunica intima and media and 3-NT immunohistochemical staining for protein nitration, as indicated. Data are presented as means ± SD (The animal number for each group is indicated in Figure 6). *, *P* < 0.05 *vs* corresponding control; #, *P* < 0.05 *vs* vehicle alcohol group. Bar = 50 μM.

In addition, treatment of alcohol-fed mice with MnTMPyP also completely prevented alcohol-induced aortic cell death, examined by TUNEL staining and oxidative damage, examined by 3-NT accumulation (Fig. 7).

## Discussion

Although the importance of Ang II/AT1 in alcoholic injury of the vascular system such as aorta has been appreciated, the causative role of Ang II/AT1 activation of NOX-mediated superoxide generation remains uncertain. Using AT1-KO mouse model, here we defined the causative role of Ang II/AT1 in the induction of alcoholic aortic oxidative stress/damage, cell death, inflammation, and remodelling; we also defined the causative role of superoxide in the induction of aortic pathogenic changes by pharmacological treatment of chronic alcohol-fed mice simultaneously with superoxide dismutase mimetic.

Endothelial dysfunction and atherosclerosis are manifested as vascular disease. Moderate alcohol consumption was reported to reduce cardiovascular diseases. However, heavy alcohol intake is almost always associated with systemic hypertension and also endothelia dysfunction and artery atherosclerosis. Studies have indicated that administration of chronic oral dose of 20% ethanol (4 g/kg) for 12 weeks, which corresponds to more than threefolds of standard alcoholic drinks per day in humans that is considered as moderate to heavy dose [21, 22], significantly increased cardiovascular events in rat model [23]. In the present study, the ethanol content (%, w/v) in the diet is 4.8 (34% of total calories) at initiation, and it gradually increased up to 5.4 (38% of total calories) for 8 weeks. In our recent study, this condition of alcoholic consumption induced significant increases in systolic and diastolic blood pressures along with the increase of systemic Ang II levels [24]. We also found that alcohol-increased blood pressure and plasma Ang II level were not affected by supplementation of superoxide dismutase mimetic MnTMPyP [24].

Ang II has been demonstrated to cause the vasoconstriction by increasing the production of superoxide via induction of NOX in the vascular wall [25, 26]. They found that the increase in blood pressure was also well correlated with activation of aortic NOX activity in ethanol-fed rat compared with control. These studies demonstrated a significant increase in NOX activity and its subunits protein expression in the vessels of chronic alcohol-treated animals, indicating the involvement of superoxide production through Ang II-mediated activation of NOX in alcohol-induced vascular injury and hypertension [23, 25, 26]. Based on these data, however, it remains unclear whether Ang II along with blood pressure induces aortic injury or vascular injury causes increase in blood pressure. In the present study, we demonstrated that alcohol induced significant increases in various pathogenic changes only in the aorta of WT mice, but not in AT1-KO mice (Figs. 5). Furthermore, we also demonstrated that treatment of alcohol-fed mice with superoxide dismutase mimetic to scavenge superoxide almost completely prevented alcohol-induced aortic pathogenic changes (Figs. 7). Our findings provide the first evidence to define the causative role of systemic Ang II in the aortic pathogenic changes induced by chronic heavy alcohol ingest.

There was a study showing enhanced production of inflammatory mediators such as TNF-α, COX-2 and MCP-1 in the aorta of chronic ethanol-fed rats compared with control [23]. Inflammation and oxidative stress are intertwined and the pro-inflammatory cytokines are capable of inducing ROS and/RNS production in the vascular system. It was assumed that these inflammatory mediators enhanced ROS and/or RNS production, to cause oxidative damage to the aortic endothelium, leading to impaired vascular relaxation and hypertension in chronic alcohol-fed rats [23]. However, our study is not in support of this notion because we demonstrated that deletion of AT1 gene or blockage of NOX-mediated superoxide generation by applying superoxide dismutase mimetic could completely prevent aortic inflammatory responses and damage (Figs. 6). Therefore, our study suggests the causative role of AT1-mediated activation of NOX-associated generation of ROS and/RNS in induction of aortic inflammatory response and remodelling.

In summary, herein we have investigated the relationship of chronic alcohol-induced inflammatory/oxidative stress signalling in the vascular endothelium with Ang II/AT1 system. We provided the first evidence to directly define the causative role of Ang II via interaction with AT1 receptor in the induction of alcoholic aortic oxidative stress/damage, inflammation, cell death and proliferation, and remodelling. More important, pharmacological treatment of alcohol-fed mice with superoxide dismutase mimetic significantly prevented aortic oxidative stress/damage, inflammation, cell death and proliferation, and remodelling. Therefore, it is suggested that angiotensin converting enzyme inhibitors/Ang II receptor blockers as well as superoxide scavenger (MnMTPyP or Tempol) are clinically useful for the intervention of chronic alcohol-induced hypertension and cardiovascular diseases.
